# Supplementary material for: Perception of social interaction compresses subjective duration in an oxytocin-dependent manner
Source: eLife. 2018 May 22;7:e32100. doi: 10.7554/eLife.32100 (PMC5963918; doi:10.7554/eLife.32100)
Supplement: Supplementary file 1. — C1-10 were chosen from the Communicative Interaction Database (Manera et al., 2010). NC1-10 were produced by cross-pairing the agents of the same gender from C1-10. [file elife-32100-supp1.docx]

**Supplementary File 1**: Description of the point-light motion sequences used in the duration judgment task. C1-10 were chosen from the Communicative Interaction Database (Manera, Schouten, Becchio, Bara, & Verfaillie, 2010). NC1-10 were produced by cross-pairing the agents of the same gender from C1-10.

| Motion Sequence | Interaction Description | Gesture | Dyadic  /Triadic | Social Motivation | Gender |
| --- | --- | --- | --- | --- | --- |
| C1 | A asks B to look at something on the ceiling, behind B; B turns 180^°^ and looks at it. | pointing | triadic | sharing | F |
| C2 |  |  |  |  | M |
| C3 | A asks B to look at something on the floor; B bends down to look at it. | pointing | triadic | sharing | F |
| C4 |  |  |  |  | M |
| C5 | A indicates to B the position of something on the floor; B bends down and picks the object up. | pointing | triadic | helping | F |
| C6 |  |  |  |  | M |
| C7 | A asks B to stand up; B, who is sitting, stands up. | gesture of the hand | triadic | giving instructions | F |
| C8 |  |  |  |  | M |
| C9 | A asks B to stop; B, who is walking, stops. | gesture of the hand | dyadic | ordering | F |
| C10 |  |  |  |  | M |
| NC1 | C1A + C9B | pointing | N/A | N/A | F |
| NC2 | C2A + C10B |  |  |  | M |
| NC3 | C3A + C1B | pointing | N/A | N/A | F |
| NC4 | C4A + C2B |  |  |  | M |
| NC5 | C5A + C7B | pointing | N/A | N/A | F |
| NC6 | C6A + C8B |  |  |  | M |
| NC7 | C7A + C3B | gesture of the hand | N/A | N/A | F |
| NC8 | C8A + C4B |  |  |  | M |
| NC9 | C9A + C5B | gesture of the hand | N/A | N/A | F |
| NC10 | C10A + C6B |  |  |  | M |
