# Supplementary material for: Perception of social interaction compresses subjective duration in an oxytocin-dependent manner
Source: eLife. 2018 May 22;7:e32100. doi: 10.7554/eLife.32100 (PMC5963918; doi:10.7554/eLife.32100)
Supplement: Transparent reporting form [file elife-32100-transrepform.docx]

***eLife’s* transparent reporting form**

We encourage authors to provide detailed information *within their submission* to facilitate the interpretation and replication of experiments. Authors can upload supporting documentation to indicate the use of appropriate reporting guidelines for health-related research (see [EQUATOR Network](http://www.equator-network.org/%20)), life science research (see the [BioSharing Information Resource](https://biosharing.org/)), or the [ARRIVE guidelines](http://www.plosbiology.org/article/info:doi/10.1371/journal.pbio.1000412) for reporting work involving animal research. Where applicable, authors should refer to any relevant reporting standards documents in this form.

If you have any questions, please consult our Journal Policies and/or contact us: [editorial@elifesciences.org](mailto:editorial@elifesciences.org).

**Sample-size estimation**

- You should state whether an appropriate sample size was computed when the study was being designed
- You should state the statistical method of sample size computation and any required assumptions
- If no explicit power analysis was used, you should describe how you decided what sample (replicate) size (number) to use

Please outline where this information can be found within the submission (e.g., sections or figure legends), or explain why this information doesn’t apply to your submission:

The sample sizes of n = 24 per group in Experiments 1-3 as well as the supplementary experiment were determined by G*Power to be sufficient to detect a large temporal distortion effect in time perception (d ≥ 0.8), at a power larger than 95%. The effect size was estimated based on an earlier study that employed biological motion stimuli and similar psychophysical testing procedures to those in the current study (Wang & Jiang, 2012). This is mentioned in the Participants section under Materials and Methods. We note that studies on time perception have commonly obtained large effect sizes (d ≥ 0.8) (Droit-Volet, Mermillod, Cocenas-Silva, & Gil, 2010; Frassinetti, Magnani, & Oliveri, 2009; Hagura, Kanai, Orgs, & Haggard, 2012; Matthews, 2015; Sadeghi, Pariyadath, Apte, Eagleman, & Cook, 2011; Zhou, Yang, Mao, & Han, 2014)*.* No statistical method was used to pre-determine the sample sizes in Experiments 4-6, as we were unaware of any oxytocin study that had examined time perception with a similar paradigm. But the sample sizes used (ns = 90, 80 and 80, respectively) are comparable to or larger than those in previous oxytocin studies (Anagnostou et al., 2012; Bartz et al., 2010; Gordon et al., 2013; Guastella et al., 2010; Parker et al., 2017). A total of 346 subjects were tested for the entire study.

**Replicates**

- You should report how often each experiment was performed
- You should include a definition of biological versus technical replication
- The data obtained should be provided and sufficient information should be provided to indicate the number of independent biological and/or technical replicates
- If you encountered any outliers, you should describe how these were handled
- Criteria for exclusion/inclusion of data should be clearly stated
- High-throughput sequence data should be uploaded before submission, with a private link for reviewers provided (these are available from both GEO and ArrayExpress)

Please outline where this information can be found within the submission (e.g., sections or figure legends), or explain why this information doesn’t apply to your submission:

Each experiment was performed once. The temporal compression effect induced by the perception of social interactions, as observed in Experiment 1, was replicated in Experiments 4 (Figure 3), 5 (after oxytocin administration, Figure 4A), and 6 (Figure 4C & 4D). The negative correlation between autistic-like tendency and the magnitude of the temporal compression effect, as observed in Experiment 4, was confirmed by Experiments 5 and 6 (before drug treatment, Figure 4). The criteria for inclusion of participants are stated in the Participants section under Materials and Methods.

**Statistical reporting**

- Statistical analysis methods should be described and justified
- Raw data should be presented in figures whenever informative to do so (typically when N per group is less than 10)
- For each experiment, you should identify the statistical tests used, exact values of N, definitions of center, methods of multiple test correction, and dispersion and precision measures (e.g., mean, median, SD, SEM, confidence intervals; and, for the major substantive results, a measure of effect size (e.g., Pearson's r, Cohen's d)
- Report exact p-values wherever possible alongside the summary statistics and 95% confidence intervals. These should be reported for all key questions and not only when the p-value is less than 0.05.

Please outline where this information can be found within the submission (e.g., sections or figure legends), or explain why this information doesn’t apply to your submission:

Statistical analysis methods are described in the Analysis section under Materials and Methods. Detailed statistical results are reported for each experiment in the Results section (Figures 2-4).

(For large datasets, or papers with a very large number of statistical tests, you may upload a single table file with tests, Ns, etc., with reference to sections in the manuscript.)

**Group allocation**

- Indicate how samples were allocated into experimental groups (in the case of clinical studies, please specify allocation to treatment method); if randomization was used, please also state if restricted randomization was applied
- Indicate if masking was used during group allocation, data collection and/or data analysis

Please outline where this information can be found within the submission (e.g., sections or figure legends), or explain why this information doesn’t apply to your submission:

Experiments 1-4 as well as the supplementary experiment do not involve group allocation. In Experiments 5 and 6, the participants were randomly allocated to one of the two drug conditions (oxytocin vs. atosiban in Experiment 5, atosiban vs. saline in Experiment 6). Drugs were administered in a double-blind manner. The information can be found in the Drug Application section under Materials and Methods.

**Additional data files (“source data”)**

- We encourage you to upload relevant additional data files, such as numerical data that are represented as a graph in a figure, or as a summary table
- Where provided, these should be in the most useful format, and they can be uploaded as “Source data” files linked to a main figure or table
- Include model definition files including the full list of parameters used
- Include code used for data analysis (e.g., R, MatLab)
- Avoid stating that data files are “available upon request”

Please indicate the figures or tables for which source data files have been provided:

All individual data and group-level statistics are plotted in Figures 2-4. We would be happy to upload the raw data files if our manuscript is found acceptable for publication in *eLife*.
